# Drug utilization research in Peru: Is real-world data available?

**DOI:** 10.3389/fphar.2022.1047946

**Published:** 2023-01-17

**Authors:** L. Yesenia Rodríguez-Tanta, Héctor Garavito Farro, Lisiane Freitas Leal, Maribel Salas, Monique M. Elseviers, Luciane Cruz Lopes

**Affiliations:** ^1^ Institute for the Evaluation of Health Technologies and Research, Social Security of Health, Lima, Peru; ^2^ Ministry of Health of Peru, Lima, Peru; ^3^ Department of Epidemiology, Biostatistics and Occupational Health, McGill University, Montreal, QC, Canada; ^4^ Daiichi Sankyo (United States), Parsippany, NJ, United States; ^5^ Perelman School of Medicine, University of Pennsylvania, Philadelphia, PA, United States; ^6^ Department of Clinical Pharmacology, Ghent University, Ghent, Belgium; ^7^ Pharmaceutical Science Graduate Course, University of Sorocaba, São Paulo, Brazil

**Keywords:** Peru (fuente), drug utilisation research, data sources, Pharmacoepidemiology, Latin America

## Abstract

**Background:** Drug utilization research (DUR) is used to provide evidence-based data to inform policies and make decisions. The aim of this study was to map and describe available data sources for drug utilization research in Peru.

**Methods:** We performed a search of data sources providing information on medication use on the website of governmental organizations. We also conducted a literature review using PubMed, LILACs, and BVS. Independently, researchers screened eligible data sources. Data characterization included accessibility, coverage data provider, type of data sources, and setting. We performed a descriptive analysis.

**Results:** We identified seven data sources, CENAFyT, ICI, IDI (SISMED), and ENSUSALUD from MINSA, and CRI-ESSALUD, SGSS/ESSI, and ENSSA from ESSALUD. These presented information on adverse drug reactions (*n* = 2), drug consumption, and distribution (*n* = 2), prescription and drug dispensing (*n* = 1), and surveys addressed to medication users (*n* = 2). ENSUSALUD was the only data source publicly available. VIGIFLOW and ENSUSALUD have a national granularity from the public and private sectors. The setting of the data sources was both hospital and ambulatory care. Two data sources have individual-level data on adverse drug reactions and one on prescriptions. Four studies on drug utilization research in Peru were derived from ENSUSALUD.

**Conclusion:** In Peru, few data sources are available for drug utilization research. There is an increased need to monitor medications for decision-making purposes. Local and international initiatives and partnerships of the government with academic institutions and the private sector might be a good strategy to increase the transparency of health data and for supporting decision-making using drug utilization research.

## Introduction

Medications are essential to improve individual patient care and public health worldwide. However, inappropriate drug use, particularly, overmedication and self-medication might lead to adverse drug reactions (ADR) (Ahmed et al., 2014; Berreni et al., 2015; Rajan et al., 2019), resulting in non-adherence problems, increase morbidity, mortality, and negative socio-economic consequences (Formica et al., 2018). For these reasons, the evaluation of the process of prescribing, dispensing, and consumption of medicines through Drug Utilization Research (DUR), particularly in low- and middle-income countries, is fundamental to informing policies and make informed decisions from health, economic and social perspectives (Massele et al., 2015).

DUR began in the 1960s and, since then, it has been progressively demanded by health authorities, consumers, and payers. Many countries have developed DUR and are taking advantage of its usefulness to improve access to medicines and to evaluate their impact on the benefit-risk in the real-world setting (Bergman, 2006; Linnér et al., 2020; Monica, 2020; Ontario Ministry of Health, 2020). European countries pioneered DUR with important results in clinical and regulatory settings. In the United States, DUR has contributed to improving the quality of patient care through effective drug management (Sabaté et al., 2014; Wettermark et al., 2016). DUR may also guide healthcare providers (HCP) on the rational use of drugs and promote their cost-effectiveness. However, DUR progress has not been the same across the globe due to multiple factors (World Health Organization, 2003). Indeed, a study that compiled information on drug data sources in Latin American (LA) found 124 potential data sources for DUR from nine countries, being Brazil and Argentine the countries with the major number of data sources. Unfortunately, accessibility to those databases is limited and only aggregate-level data was available, given this situation, the DUR implementation is scarce in LA (Lopes et al., 2022).

**FIGURE 1:**
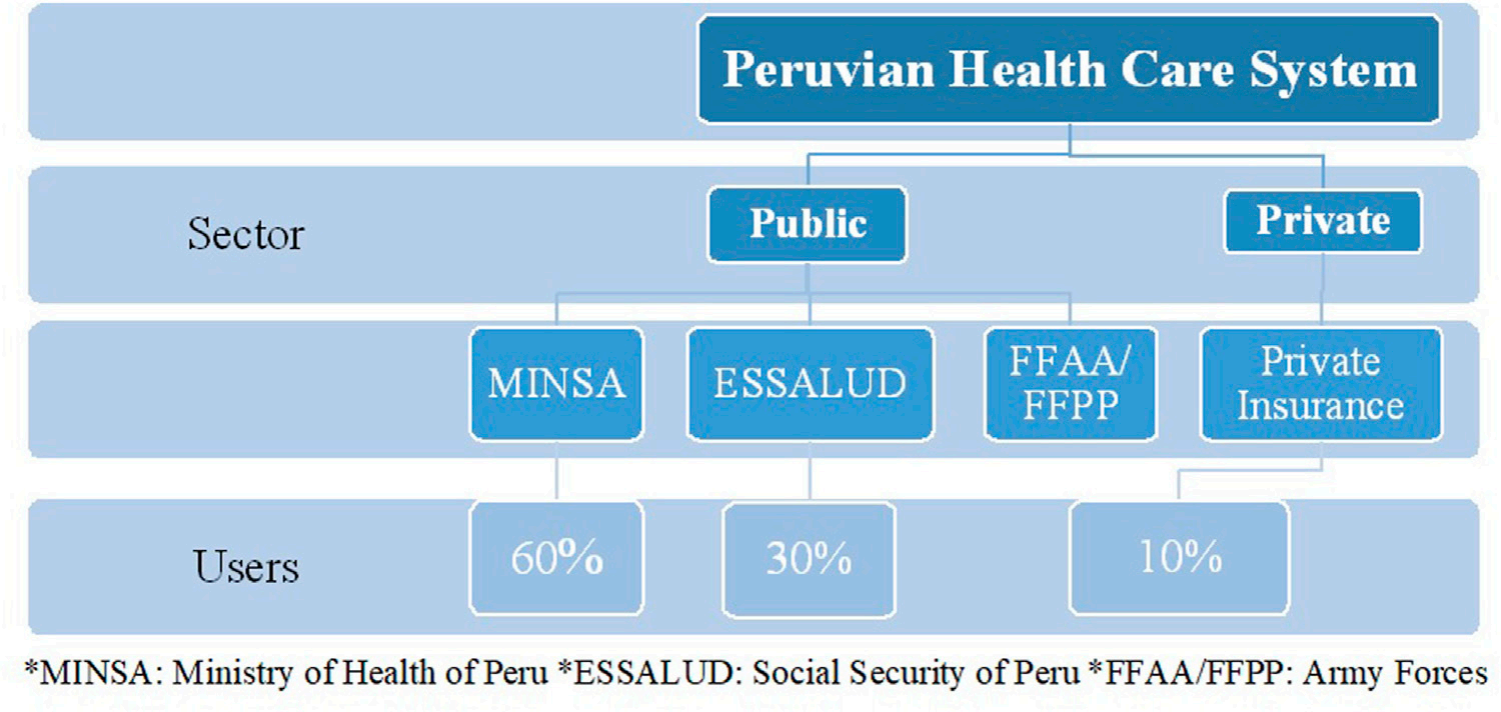
Peruvian Healthcare System Structure. *MINSA, Ministry of Health of Peru; *ESSALUD, Social Security of Peru; *FFAA/FFPP, Army Forces.

Additionally, in many LA countries, the lack of identification of data sources with information about medicines use and the decentralized healthcare systems are the main barriers to conduct DUR (Sales et al., 2020). Peru has a significantly fragmented healthcare system divided into five entities: the Ministry of Health (MINSA) which covers almost the majority of the population (60%), the Social Security of Health (ESSALUD) covering 30%, and the Armed Forces (FFAA), Peruvian National Police (PNP), and the private sector that cover the remaining part of the population (Arroyo et al., 2011) (Figure 1). Each entity has, therefore, its own information, making it difficult to implement DUR at a national level. Consequently, the General Directorate of Medicines Supplies and Drugs (DIGEMID) of the MINSA, which is responsible for national drug policy, has no access to data concerning drug utilization which limits the improvement of making decisions based on real-world evidence.

In Peru, pharmaceutical products and medical devices are regulated by the DIGEMID based on technical information on efficacy, safety, and quality. Once products are in the market, purchases of medicines are centralized and distributed at the national level and also can be acquired locally by regional authorities (Coronado and Espinoza, 2014). The government assures price regulation for medicines only in the public sector. In addition, Peru, as part of the National Drug Policy, has its own National List of Essential Drugs (PNUME) based on the model list provided by the World Health Organization (WHO). PNUME is managed by MINSA with the aiming to cover the priority healthcare needs of the population (Petitorio, 2020). Recently, the introduction of health technology assessment (HTA) and the development of evidence-based clinical guidelines in the Peruvian public sector are improving drug policies and access to medicines. Ever since ESSALUD deployed a decision-making process based on HTA to cover new medications or medical devices, the efficient use of resources and the transparency of the approval process have been enhanced. Indeed, the average of money invested to better the patient’s access to new technologies has decreased from S/133, 270.00 in 2011 to S/. 47,779.00 in 2019 *per capita* (Peralta et al., 2022). However, the increase in pharmaceutical expenditure and the knowledge on how medicines are prescribed and used across insurance schemes through DUR are limited.

Identifying drug data sources is relevant to perform DUR which in turn, may contribute to public health from medical, social, and economic perspectives (Sacristán and Soto, 1994; Wettermark et al., 2016; Peralta et al., 2022). Some initiatives to map drug databases have been developed, especially in Europe, to help stakeholders make informed decisions to promote the rational use of medicines (Eriksson and Ibáñez, 2016; ENCePP, 2022). To our knowledge, no mapping has been conducted until now to describe the existence of data sources for DUR in Peru, where the demand for medications is increasing. Therefore, the aim of this study is to map and describe data sources accessible for Drug Utilization Research in Peru by conducting a review of public Peruvian Health Institutions and scientific databases. We believe this manuscript might help inform Peruvian healthcare policy and enhance drug policies worldwide.

## Methods

The methods were based on the DASDUR-LATAM Study which was sponsored by ISPE (Lopes et al., 2022). DASDUR-LATAM is a cross-sectional comparison study that aimed to compile an inventory of available national drug data sources from nine countries.

### Design

This was a cross-sectional study in which an expert network of health national data providers and academic specialists on DUR created an inventory of data sources for DUR in Peru.

### Type of data sources (eligibility criteria)

We defined a data source for DUR as a database with information on medicines including volume, price, dose, or safety information (for example, notification of adverse drug reactions). Included drug data sources of large jurisdictions or from organizations multi-sited that serve a large population, supported by governmental organizations. Considered jurisdictions were at the national and/or regional level. We did not include in this inventory data sources from health insurance companies, sickness funds, and private organizations (e.g., IQVIA) because payment to access them is required. Data sources containing a mix of data from the public and private health sectors were included.

### Search strategy

First, we carried out a search of data sources providing information on access, use, and safety of drugs on the websites of Peruvian institutions such as the General Directorate of Medicines Supplies and Drugs (DIGEMID) and the National Superintendence of Health of Peru (SUSALUD) both from the Ministry of Health of Peru (MINSA) and Peruvian Social Security (ESSALUD), as well as academic institutional repositories from the main Peruvian universities.

Second, we performed a scientific literature review on PubMed, SciELO, and BVS to identify studies that used such data sources in Peru. We also searched for gray literature (thesis, unpublished studies, abstracts from conferences and meetings), and used a citation manager to import references (see Supplementary Appendix SA). We used the following combined keywords “drug data sources,” “drug utilization,” “pharmacovigilance,” and “Peru.” There was no limitation either by type of study or language. Both searches were conducted in July 2019 and updated in February 2020.

### Screening process

Working in pairs and independently two researchers (YR, HG), conducted a screening and reviewed the potentially eligible data sources. The divergences in the usefulness of each database for DUR were discussed during the monthly meeting with the coordinator of the project (LCL) for consensus.

### Data extraction and analysis

Once eligible data sources were selected from Peruvian health institutions’ websites and studies using data sources were retrieved, two reviewers independently extracted the following information:

i. Data provider custodian, steward;
ii. Accessibility (publicly available and convenient; restricted pre-authorized protocol only access; access limited to or dependent on country-specific legislation; available only to researchers working at each institution; the process for obtaining data is not clear without general regulation; not accessible anyway) at which level the data was generated (whole-sellers, pharmacy, physician, others);
iii. Healthcare setting (hospital, ambulatory care, both);
iv. Years coverage;
v. Geographic granularity (national, regional, municipal, organization multi-sited, other);
vi. Type of data (aggregate or individual level);
vii. Sector data source (public, private, or both).

In some cases, we contacted data sources to ensure accuracy and improve completeness. We analyzed data collected descriptively and summarized it in tables. In case of disagreement, the coordinating team (LCL, ME, MS) was consulted for consensus.

## Results

### Drug database selection

**FIGURE 2:**
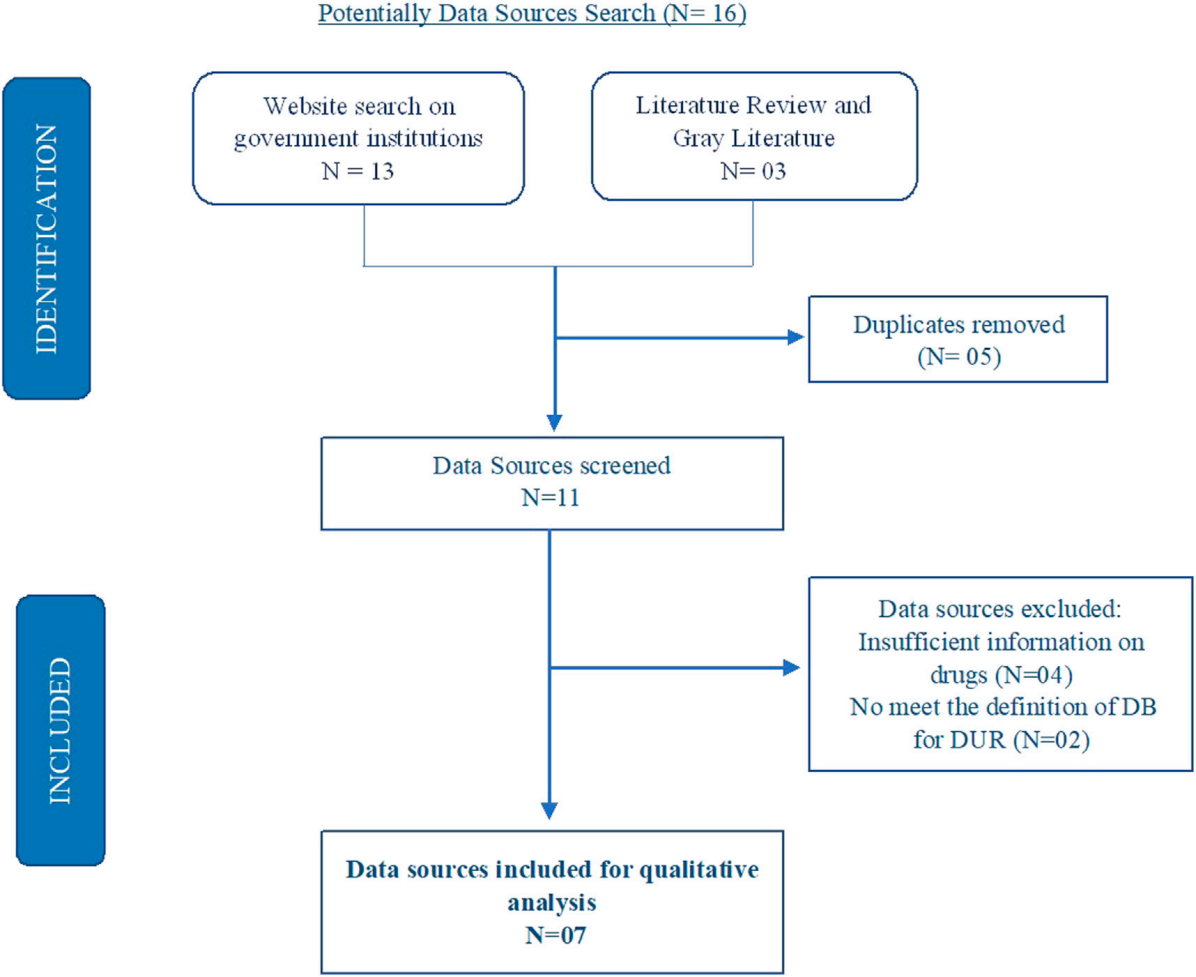
Drug data sources search flowchart. Potentially Data Sources Search (*N* = 16).

We identified a total of seven drug data sources from Peruvian Health institutions for the qualitative analysis (Figure 2).

- Pharmacovigilance data base of the National Center of Pharmacovigilance and Technovigilance (CENAFyT)
- Data Base of ADR of the Reference Center of Pharmacovigilance and Technovigilance of EsSalud (CRI-ESSALUD);
- Hospital Management System of EsSalud (SGSS/ESSI);
- National Socioeconomic Survey of Access to Health (ENSSA)
- National Survey on User Satisfaction of Health Services (ENSUSALUD) (Su perintendencia Nacional de Salud, 2016);
- Consumption of Medications and Medical Devices (ICI) (Ministerio de Salud, 2022)
- Distribution of Medications and Medical Devices (IDI) (Ministerio de Salud, 2022)

From the systematic search performed in PubMed, SciELO and BVS, we initially identified a total of 305 studies and after screening the full text manually, we found five studies, four used the National Survey on User Satisfaction of Health Services (ENSUSALUD 2015–2017) and the other the Drug Integrated System (SISMED) of the Ministry of Health of Peru (Supplementary Figure S1).

The drug data sources identified from the literature review on PubMed, SciELO, and BVS were ENSUSALUD and ICI. Five studies were conducted using these databases (Hodgkin et al., 2014; Urrunaga-Pastor et al., 2015; Mezones-Holguín et al., 2016; Hernández-Vásquez et al., 2018; Rojas-Adrianzén et al., 2018). From the gray literature search, we found CENAFyT. This data source was used in a bachelor thesis that aimed to assess the quality of ADR reporting from marketing authorization holders (MAH) using VIGIGRADE (Condori Quito and Hernandez Loli DM de los, 2018).

### Drug data sources characteristics

CENAFyT and CRI-ESSALUD contained information on ADR at the individual level. SGSS/ESSI has data on prescriptions and pharmacy dispensation from medical centers, ENSSA and ENSUSALUD provide data from surveys addressed to users at the aggregate level. Finally, ICI and IDI, drug data sources that comprised the Integrated Supply System for Medicines and Medical-Surgical (SISMED), display data on drug consumption and distribution (see Tables 1, 2).

**TABLE 1:** General description of data sources for DUR in Peru.

Data source acronym	Full name of the data source	Description	Custodian	Website	Published studies using the data source
CENAFyT	National Center of Pharmacovigilance and Technovigilance	It has its DB containing information on ADRs at individual-level collected from HCP and MAH using “eReporting” provided by Uppsala Monitoring Centre (UMC)—WHO	DIGEMID—MINSA	Not public	01
CRI-ESSALUD	Reference Center of Pharmacovigilance and Technovigilance of EsSalud	It has its DB containing information on ADRs at an individual-level reported by HCP from 390 medical centers of ESSALUD since 2015	IETSI—ESSALUD	Not public	No
SGSS/ESSI	Hospital Management System of EsSalud	Contains information on pharmacy dispensation, prescriptions at the individual level and medication stock from each medical center of ESSALUD	ESSALUD	No public	No
ENSSA	National Socioeconomic Survey of Access to Health	DB containing information from a survey aimed at roughly 63,000 patients covered by ESSALUD in 2015. It provides data about demographic characteristics, health services knowledge, chronic conditions, and medication consumption	ESSALUD	No public	01
ENSUSALUD Su perintendencia Nacional de Salud. (2016)	National Survey on User Satisfaction of Health Services	DB including information from face-to-face questionnaires addressed to HCP and users of medical centers and pharmacies to know their perspectives on the Health System of Peru. It provides data about demographic characteristics, health services knowledge, chronic conditions, purchasing, and consumption of medicines. It is performed annually since 2014.	SUSALUD—MINSA	http://portal.susalud.gob.pe/blog/encuestas-de-satisfaccion-a-nivel-nacional-ensusalud-2016/ (There is a link per each year)	04
ICI Ministerio de Salud (2022)	Consumption of Medications and Medical Devices of the SISMED	SISMED compiles reports of drug inventories sent by medical centers and institutional pharmacies of the MINSA at a national level. ICI table of the SISMED provides the detail of the stock, sales and number of dispensed products. The average consumption per medication and the stock on a monthly-basis per each center can be calculated.	DIGEMID—MINSA	https://appsalud.minsa.gob.pe/consolida/portalsismed/RepPrecioMedicamento.aspx	01
IDI Ministerio de Salud (2022)	Distribution of Medications and Medical Devices of the SISMED	IDI table contains the details of the distribution of medical products and medical devices at MINSA and provides information on the departure and arrival of products (dates and quantities) and is updated on a daily basis.	DIGEMID—MINSA	https://appsalud.minsa.gob.pe/consolida/portalsismed/RepPrecioMedicamento.aspx	No

DIGEMID, General Directorate of Medicines Supplies and Drugs; MINSA, Ministry of Health of Peru; IETSI, Institute of Health Technology Assessment; ESSALUD, Peruvian Social Security; SUSALUD, National Superintendence of Health of Peru; SISMED, Integrated Supply System for Medicines and Devices; DB, Database; ADR, Adverse Drug Reaction; HCP, Healthcare Providers.

**TABLE 2:** Characteristics of data sources identified for DUR in Peru.

Data sources	VIGIFLOW	CRI-ESSALUD	SGSS/ESSI	ENSSA	ENSUSALUD	ICI	IDI
Accessibility							
Publicly and conveniently accessible on line					X		
Restricted pre-authorized protocol-only access							
Access limited to or dependent on country-specific legislation—Freedom of Information Act							
Available only researchers working in the institution (It is only people that is from the institution that provide the DB)							
The process for obtaining data is not clear, without general regulation	X	X	X	X		X	X
Not accessible any way/Data not available for public use							
Geographic granularity							
National	X				X	X	X
Regional (province, state, more than one city)							
Municipality (one city)							
Organization multi-sited		X	X	X			
Other (specify)							
Sector of data source							
Public health system		X	X	X		X	X
Private sector							
Both	X				X		
Type of data source							
Wholesaler							X
Pharmacy records			X			X	X
Patient records				X			
Other (specify)	ADR notifications (ICSR)	ADR notifications (ICSR)	Prescriptions and consumptions	Survey	Survey	Consumptions and stock	Distributions
Setting of data source							
Ambulatory				X			
Hospital							
Both (possible to separate)	X	X	X		X	X	X
Both (not possible to separate)							
Other (specify)							
Years coverage							
SINCE	2006	2015	2011	2015	2014	2010	2010
Type of data							
Aggregate				X	X	X	X
Individual-level	X	X	X				

ADR, Adverse drug reaction; ICSR, Individual case safety report.

Regarding the institutional origin, CENAFyT, ENSUSALUD, ICI, and IDI were found on the MINSA websites. It is important to highlight that CENAFyT is a division of the DIGEMID of the MINSA. On the other hand, SGSS/ESSI, ENSSA, and CRI-ESSALUD drug data sources belong to ESSALUD.

In terms of accessibility, ENSUSALUD was the only data source publicly available. The MINSA through SUSALUD has included questionnaires denominated ENSUSALUD from 2014. These surveys are applied annually to health services users (healthcare providers and patients) to assess the level of satisfaction. The process for obtaining data from these data sources was not clear for the other drug data sources.

CENAFyT of the DIGEMID and ENSUSALUD were the only data sources with national information from the public and private sectors. DIGEMID as a regulatory agency is responsible to conduct the Peruvian System of Pharmacovigilance through the collection of ADRs notification not only from MINSA, ESSALUD, FFAA, and PNP, but also from the pharmaceutical industry. Likewise, ENSUSALUD is conducted annually to collect information on private sector users also (pharmaceutical companies and private pharmacies and drugstores).

CRI-ESSALUD, SGSS/ESSI, and ENSSA were multi-sited organizations. CRI-ESSALUD has national granularity but it only collects individual case safety reports from healthcare providers (medical doctors, pharmacists, nurses, and dentists) working at 400 medical centers of ESSALUD. Furthermore, both data sources, ICI and IDI, included important drug information at the national level but only from healthcare institutions of the MINSA.

Regarding the setting, CENAFyT, CRI-ESSALUD, SGSS/ESSI, ENSSA, ICI, and IDI incorporate information on hospital and ambulatory care. Most of the drug data sources had less than 10 years of coverage with exception of CENAFyT.

## Discussion

In this study, we identified seven data sources that potentially might be useful to perform DUR. To our knowledge, this is the first mapping to identify data sources for DUR in Peru. These data sources included institutions under the control of the Government (MINSA and ESSALUD) and constitute information on ADR, prescription, dispensing, consumption, and distribution of medication. However, they have limited available data for DUR and many restrictions to access. The National Survey on User Satisfaction of Health Services (ENSUSALUD) was the only data source publicly available. ENSUSALUD is carried out annually by SUSALUD of MINSA since 2014 to assess the performance of the Healthcare System in Peru. This survey targets healthcare providers and users (e.g., patients), and data is freely available on the website of SUSALUD. This data source was the most frequently used for DUR purposes. As a result of the review, we found four studies that aimed to evaluate factors associated with self-medication and the purchase of medications without prescription (Urrunaga-Pastor et al., 2015; Mezones-Holguín et al., 2016; Hernández-Vásquez et al., 2018; Rojas-Adrianzén et al., 2018).

Regarding the existing drug data sources in Peru, four were at population-level information. Aggregate-level data, such as sales data, can play an important role to evaluate the impact of the pharmaceutical policies and interventions, however, individual-level data might enhance the insight of DUR because it is possible for researchers to visualize drug distribution (incidence, prevalence, drug duration and patterns of drug use over the time), assess temporal sequences, identify problems related to prescription (cascade prescriptions due to adverse drug reactions), etc. (Sacristán and Soto, 1994; Hallas, 2005). Unfortunately, we only identified three drug data sources at an individual-level with limited information on drug and patient characteristics. Since research on DUR is relevant to help inform healthcare policy, we believe that the government should deploy activities to increase the collection of drug utilization and clinical outcomes information at a patient-level, particularly in important settings such as hospitals, physician practices, pharmacies, and drugstores.

Few studies have been conducted to identify data sources available for DUR in LA. Justo et al. (2019) recently published an analysis to characterize and evaluate data sources and their uses in Argentina, Brazil, Colombia, and Chile. The authors identified a total of 407 unique databases, but the fragmentation of the healthcare system and the lack of resources and infrastructure are the major limitations to collecting interoperable data from many institutions and organizations (e.g., the government, providers, insurers, etc.). In addition, the authors describe that real-world evidence (RWE) is not used for health technology assessment (HTA) purposes, but mostly for pharmacovigilance (Justo et al., 2019). In 2014, Gregory (2014) performed a systematic review of the literature to examine administrative and clinical data for 10 major LA countries. They found Brazil, Mexico, Argentina, and Chile as having contributory information on data sources for RWE. However, given the low quality of databases, well-designed prospective studies should be conducted to improve RWE (Gregory, 2014).

The scarce number of drug data sources in Peru might be due to several factors. First, Peru currently has one of the lowest public spendings on health at 2.2% of Gross Domestic Product (GDP) among LA countries, and it is estimated that the public sector accounts for only 30% of the expenditure on medications. Second, the Healthcare System is fragmented which represents a limitation on the collection of data on access, distribution, and use of medicines. Third, administrative and care patient data coming from the four public health institutions (MINSA, ESSALUD, FFAA, and PNP) are not systematically recorded and the little information available is not integrated. Fourth, nearly 31% of patients have limited access to medicines although the Peruvian Government has deployed strategies to improve universal health coverage (Velásquez et al., 2016). Finally, decision-makers in Peru, as in many LA countries, maybe not be acquainted with the importance of DUR (Moscou et al., 2016). In consequence, Peruvian authorities are limited to making evidence-based decisions based on the real use, safety, effectiveness of pharmaceutical products already marketed which can have a negative impact on the cost of technology acquisition, reimbursement, efficient use of resources and the transparency of processes in clinical settings.

Using one or a few drug data sources may be weaker and hence is not actually representative of the real-world setting of drug use. Addressing a limited number of drug data sources in Peru requires the involvement of different institutions and organizations, mainly the MINSA and ESSALUD, and to be more economically resourced. Although public institutions like DIGEMID of the MINSA play key roles in collecting population-level drug data (distribution, consumption, and individual case safety reports); the major disadvantage is the fact that those data are not linked. Record linkage enables researchers to have more extensive prescriber and patient information to study drug utilization (Sacristán and Soto, 1994). In consequence, the main challenges are to integrate data across entities to obtain a robust data system and to have a standard process to extract information to best answer any DUR research. In addition, more work is needed to improve the quality, accessibility, and transparency of drug data sources particularly, for research purposes. Collaboration among academia, stakeholders, government and the private sector might be a good strategy to improve drug data sources and research.

There is an increased need to develop DUR in Peru due to the increased demand for medications (UgarteUbilluz, 2019). Currently, there is limited and incomplete information on drug use due to various factors, such as inadequate drug information, HCP with basic training on drugs, prescribing and dispensing activities influenced by socio-cultural patient demands and drug promotion, and the weakness of Peruvian drug policies (Paredes et al., 1996; Maldonado et al., 2002; Bustamante Robles et al., 2012; Florián-Castro, 2018). This situation derives from issues, such as over-prescription (Bustamante Robles et al., 2012), medication non-adherence, drug misuse in vulnerable populations [e.g., polypharmacy in the elderly, off-label use of medications in pregnancy, overuse of antibiotics in children (Ecker et al., 2011; Ecker et al., 2013; Fernandez-Arias et al., 2014; BojórquezGiraldo et al., 2017; Leyva-Moral et al., 2019; Rueda et al., 2019), self-medication (Hernández-Vásquez et al., 2018), purchasing medications without medical prescription, especially in poor regions with bounded access to public healthcare services in Peru (Urrunaga-Pastor et al., 2015; Hernández-Vásquez et al., 2018)], which lead to negative outcomes in patient’s health. Ideally, these issues should be characterized and quantified through DUR.

It is important to consider that developing DUR might provide essential information on the use of medications among Peruvians from social, cultural, and economic perspectives. Furthermore, DUR might be the gateway to better-informed decisions and provides an excellent opportunity to set priorities for healthcare budgets (Sales et al., 2020). These priorities might complement HTA through the improvement of the rational use of medications and promoting pharmaceutical policies for the wellbeing of patients.

Our study has some limitations. To begin with, details on special data sources description (e.g., number of variables and observations) could be missing since we did not access to the data sources themselves because special permission is required. In addition, we could not validate the quality of information described in each drug data source identified since we were only focused on the identification of their characteristics and their convenience for DUR in Peru. Finally, since the literature review performed in this study is not a systematic review, it is possible that our search did not identify all available evidence.

When it comes to the strengths, the major asset is that the description of the characteristics of most data sources was based on information provided directly by database owners and workers of the MINSA and ESSALUD. In addition, in spite of the limitations of the literature review performed besides the electronic searching on websites of Peruvian health institutions, we consider that this process enabled us to identify data sources used for secondary analysis in some studies.

## Conclusion

Few data sources are available for DUR in Peru. There is an increased need to develop DUR in Peru due to the high demand for medications. The main challenges are to increase the collection of drug utilization and clinical outcomes information at a patient-level, integrate data across institutions, and to have a standard process to extract information. In addition, more work needs to be done to improve the accessibility, quality, and transparency of drug data sources particularly, for research purposes. Collaboration between academia, the private sector, the government, international institutions, and other stakeholders might be a good strategy to improve drug utilization research in Peru. DUR results might be important not only to implement strategies to deliver rational drug policy but also to provide information on the efficiency of drug use, which may be useful to set priorities for financial healthcare budgets. In addition, the availability of robust drug data sources enables researchers to use data to perform cross-national comparative drug utilization research and to enhance drug policies worldwide.

## Data Availability

The raw data supporting the conclusion of this article will be made available by the authors, without undue reservation.
