# Supplementary material for: Drug utilization research in Peru: Is real-world data available?
Source: Front Pharmacol. 2023 Jan 17;13:1047946. doi: 10.3389/fphar.2022.1047946 (PMC9888663; doi:10.3389/fphar.2022.1047946)
Supplement: Supplementary file 1 [file Presentation1.pdf]

SUPPLEMENTARY MATERIAL

Literature review

We developed and implemented a searching strategy using MEDLINE (PubMed), Scientific Electronic Library Online (SciELO) and The Virtual Health Library (BVS). The literature search strategy (((((((drug utilization) OR drug utilisation) OR pharmacoepidemiology) OR drug rational use) OR drug utilization study) OR pharmacovigilance) OR drug use) AND Peru))))). There was no limitation either by type of study or language.

A total of 305 studies were identified in PubMed, SciELO and BVS. We obtained 13 articles after reading the title and abstract, eliminating duplicates and irrelevant articles. After screening the full text manually, 9 studies were excluded because they did not report Peruvian drug data sources (n=04) and were focused on individual-patient information (n=05). Finally, we found five studies, four used the National Survey on User Satisfaction of Health Services (ENSUSALUD 2015- 2017) and the other the Drug Integrated System (SISMED) of the Ministry of Health of Peru (Fig. Nº1). The first study identified was published in 2014 and corresponds to a cross-sectional study that aimed to describe the availability of psychotropic medications for the treatment of mental disorders analyzing information of SISMED (26). Other studies were secondary analysis of the ENSUSALUD (2014-2016 surveys) that aimed to evaluate self-medication in Peru from different perspectives and the prevalence of ineffective access to drugs (22–25). From the gray literature, we identified a thesis from the Pharmacovigilance Database of DIGEMID that aimed to assess the quality of ADR reporting from marketing authorization holders (MAH) using VIGIGRADE.
